# Gene Editing Approaches against Viral Infections and Strategy to Prevent Occurrence of Viral Escape

**DOI:** 10.1371/journal.ppat.1005953

**Published:** 2016-12-08

**Authors:** Martyn K. White, Wenhui Hu, Kamel Khalili

**Affiliations:** Department of Neuroscience, Center for Neurovirology and Comprehensive NeuroAIDS Center, Lewis Katz School of Medicine at Temple University, Philadelphia, Pennsylvania, United States of America; University of Pittsburgh, UNITED STATES

## Introduction

Powerful new gene editing techniques promise groundbreaking opportunities for novel therapeutic options to important illnesses, including cancer, genetic disorders [1], and viral infections [2]. These techniques include zinc finger nucleases (ZFN) [3], transcription activator-like effector nucleases (TALEN) [4], and clustered regulatory interspaced short palindromic repeat (CRISPR)-associated 9 (Cas9) [5, 6]. In particular, CRISPR/Cas9 provides an effective, highly specific, and versatile tool applicable to important human viruses, including HIV-1 [7]. CRISPR/Cas9 is elegant and simple compared to ZFN and TALEN because it uses one or more guide RNAs (gRNA), which are simple to produce and specifically target any sequence in an adaptable and flexible way for different targets, such as viral genes, by changing the gRNA sequence [8].

Cas9 cuts both strands of the DNA target, resulting in a double-strand break (DSB), usually repaired by nonhomologous end-joining (NHEJ), an error-prone arm of the DNA repair pathway that introduces insertions and deletions (InDels) at the break site. While InDel introduction is quite rare, the products of accurate repair are targets for recleavage by CRISPR/Cas9, whereas InDel products are not, and, hence, InDel products accumulate with repeated cleavage cycles [9, 10].

In this short review, we discuss CRISPR/Cas9 in combating human viruses by specifically targeting and disrupting essential viral genes. We will also discuss generation of viral escape mutants resistant to Cas9/gRNA and how this may be overcome.

## Application to Human Viruses

Genetic editing applications, including CRISPR/Cas9, can disrupt both episomal and integrated DNA viruses and can be applied to several human viruses [2], including papillomaviruses HPV16 and HPV18 [11–13], hepatitis B virus (HBV) [14–17], Epstein-Barr virus (EBV) [18, 19], HIV-1 [7, 20–27], polyomavirus JC (JCV) [28], Herpes simplex virus-1 [29, 30], and other herpesviruses [30].

HPV16 and HPV18 are DNA viruses that cause cervical carcinoma through viral proteins E6 and E7. CRISPR/Cas9 targeting HPV E6 and E7 genes is effective with HPV-transformed cell lines and is potentially applicable to effective clinical therapy for HPV-associated tumors [12–14]. HBV is a DNA virus and causes acute and chronic liver infections and hepatocellular carcinoma (HCC). CRISPR/Cas9 can disrupt HBV in vitro and in vivo and has potential in the eradication of persistent HBV infections [11, 13, 15, 16]. EBV is a herpesvirus and causes Burkitt’s lymphoma and nasopharyngeal carcinoma. CRISPR/Cas9 can ablate expression of EBV genes and is feasible as an approach to EBV infection [18, 19].

HIV provirus permanently integrates into the host genome and becomes a persistent viral reservoir with potential to reactivate and cause disease anew, making HIV-1/AIDS a chronic, lifelong infection refractory to the immune system and antiviral drug regimens. CRISPR/Cas9 allows the excision of segments of integrated proviral DNA in different latently infected cell types by targeting sequences within the HIV-1 long terminal repeat (LTR) flanking the provirus and allowing complete provirus excision [20–22, 24–26]. These experiments were performed with T cells [20], microglia, promonocytic T cells [21], primary CD4+ T-cells cultured ex vivo [22], induced pluripotent stem cells [24], HEK293T cells [25], and Jurkat cells [26], indicating that the ability of CRISPR/Cas9 to excise HIV provirus is not restricted by cell-type–specific factors. While the fate of the eliminated DNA arising after excision of segments of integrated proviral DNA is not known for certain, we tend to think that after excision from the genome, it will be degraded. Thus, there is no published evidence that extrachromosomal double-stranded circles resulting from excision can integrate back into the chromosomal DNA. For example, we recently used CRISPR/Cas9 gene editing to eliminate HIV-1 genomes from human T-lymphoid cells and comprehensively assessed the HIV-1 eradicated cells by whole-genome sequencing to rule out any off-target effects [22]. No reintegrated viral DNA was found in this study.

The CRISPR/Cas9 approach has also allowed uninfected cells to be prophylactically protected against HIV infection [21, 22]. CRISPR/Cas9 was used in two murine models in which tail-vein or intraperitoneal injection of transgenic animals with virus vector expressing Cas9 and a multiplex of gRNAs resulted in the cleavage of integrated HIV-1 DNA provirus in many tissues, indicating proof-of-concept for in vivo eradication of integrated HIV-1 DNA by CRISPR/Cas9 [23]. Kaminski et al [27] personalized CRISPR/Cas9 activity by placing the Cas9 gene under the control of an HIV-1 promoter, which is activated by the HIV-1 Tat. Functional activation of CRISPR/Cas9 by Tat occurred during HIV-1 infection, resulting in the excision of a designated segment of integrated HIV-1 proviral DNA and consequently suppressing viral expression [27]. Thus, regulated expression of Cas9 by Tat provides a novel and safe strategy for ablation of HIV-1 at an early stage of acute infection and silent proviral reactivation in latently infected cells.

In addition to CRISPR/Cas9, the Cre recombinase gene editing approach can be used against HIV-1. This approach uses Cre recombinase from bacteriophage P1 to carry out site-specific recombination between two DNA recognition sites, known as LoxP sites, allowing precise manipulation of genomes, and has been used widely in mouse genetics [31]. Cre target specificity can be manipulated to generate novel site-specific recombinases via directed evolution [32] using substrate-linked protein evolution (SLiPE), which places the recombination target site of interest adjacent to the recombinase coding region, allowing DNA molecules with a successful recombinase coding region to be marked on the linked substrate DNA and recovered from the background of unsuccessful recombinases by PCR [32]. SLiPE has been employed to evolve recombinase-recognizing sequences within an HIV-1 LTR to efficiently excise integrated HIV-1 provirus from latently infected cells [33]. LTR-specific recombinase (Tre-recombinase) is proven to be a promising tool for excision of HIV-1 provirus from infected cells [33, 34]. Hauber et al. [34] reported conditional expression of a Tre-recombinase from self-inactivating lentivirus in HIV-infected cells, which resulted in HIV-1 provirus excision that was effective in vivo in humanized Rag2^-/-^,γ^-/-^ mice engrafted with either Tre-transduced primary CD4^+^ or CD34^+^ cells [35]. Karpinski et al. [36] used SLiPE to evolve a novel recombinase (Brec1) able to recognize a sequence present in most clinically relevant HIV-1 LTRs subtypes and strains, which excised integrated HIV-1 provirus for a number of clinical isolates of HIV-1 in vitro and in vivo, including mice humanized with cells from patients [36].

The recombinase and CRISPR/Cas9 approaches are suitable for use with either double-stranded (ds) DNA viruses or retroviruses that have dsDNA as part of the life cycle (HIV-1). However, Cas9 endonuclease from *Francisella novicida* can target RNA and inhibits the RNA virus hepatitis C [37], and it may be applicable to other human RNA viruses [38].

## The Problem of Viral Escape

Several reports have highlighted a caveat in CRISPR/Cas9 that is important to address when choosing gene editing strategy. CRISPR/Cas9 targeting of HIV-1 generated mutant viruses able to escape and replicate [39–42], as observed earlier with RNAi approaches [43–45]. One report described a mutant HIV-1 generated after ZFN therapy [39], and other reports indicated CRISPR/Cas9 gave profound suppression of HIV replication, but escape mutations were rapidly and consistently generated [40–42]. Escape mutants arose by insertions, deletions, and substitutions (InDels) located within the target site for Cas9 cleavage and are typical for DSBs repaired by NHEJ [40]. Cas9 cleavage inactivates the virus by introduction of mutations by NHEJ, but a subset of these retain viability and escape and are no longer susceptible to the original gRNA. Interestingly, while most InDels contributing to escape at non-coding regions were a single base pair, three base pair InDels were observed when the target was within an HIV-1 coding region; i.e., the InDel event may preserve the HIV-1 open reading frame but destroy the CRISPR gRNA sequence homology [42]. The occurrence of InDel escape mutations is a consequence of NHEJ DNA repair and so may also occur for any DNA virus or retrovirus and, indeed, was recently reported for the pseudorabies herpesvirus [46]. NHEJ is almost always the dominant mode of DNA repair [47, 48] and is the desired pathway of repair of CRISPR/Cas9-generated DSBs, because the purpose of CRISPR/Cas9 is to introduce mutations and inactivate viruses. It is also important to note that DSB and NHEJ repairs may have very different rates in some cells as compared to other cell types; e.g., T-cells relative to myeloid cells [49, 50]. However, as noted above, in studies of HIV-1 provirus excision, no cell-type-specific differences were observed.

## Strategies to Combat Viral Escape

**Fig 1:**
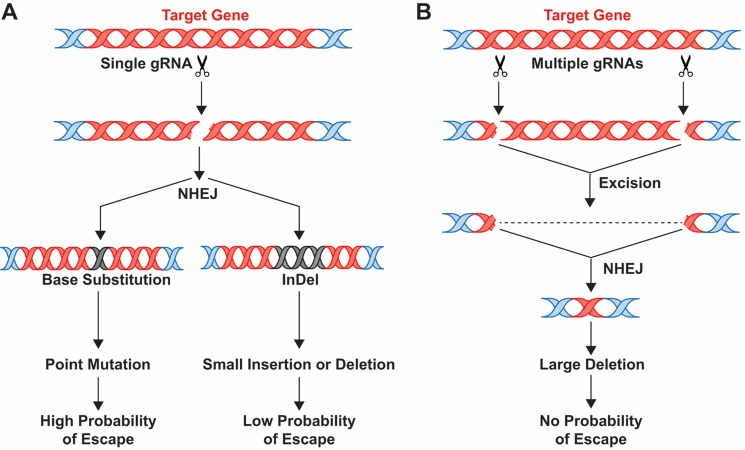
Schematic of the CRIPSR/Cas9 approach to viral inactivation and generation of escape mutants. CRISPR/Cas9 endonuclease can be used to cut a viral genome within an essential target gene using either a single gRNA (**A**) or multiple gRNAs (**B**). With a single gRNA, a base substitution or InDel mutation may occur on repair of the double-strand DNA break by error-prone non-homologous end-joining (NHEJ), which inactivates the virus. However, it is also possible that cleavage at a single site and error-prone NHEJ may also allow the occurrence and selection of escape mutants (**A**). However, when multiple gRNAs are used, this allows the creation of a large deletion with no protein produced and no possibility of escape mutations occurring (**B**).

Viral escape is not insurmountable if an appropriate choice of gene editing strategy is adopted. InDels introduced by NHEJ following CRISPR/Cas9 cleavage can cause frameshifts or premature stop codons, disrupting the target gene and abrogating its function and viral viability. If a unique locus is targeted, there is a significant possibility that InDels will be generated that allow viral escape; however, if multiplex gRNAs are employed, then the probability of this is greatly reduced, because the chances of two or more viability-conferring mutations are much less, as seen for mutiplex RNA interference (RNAi) [51]. Alternatively, if two gRNAs produce DNA breaks, allowing excision of a large section of DNA, this will permanently prevent the occurrence of escape mutations, as shown in Fig 1. Several studies have demonstrated the strong suppression of HIV-1 using a multiplex approach [21–25]. Table 1 summarizes recent studies [52–75] on the use of CRISPR/Cas9 technology for editing several human viruses using single and multiplex gRNAs, resulting in the introduction of InDels and/or excision of the segment of the viral genome.

**Table 1:** Human viral targets of CRISPR/Cas9.

Virus	Target	gRNAs		Inhibitory effects		Reference
		Monoplex (InDel)	Multiplex (InDel/excision)	In vitro	In vivo	
	-IFI16	-	+	+++	ND	[52]
						
HSV-1	-Torsin A and B	+	-	+++	ND	[53]
	LAP1, LULL1					
						
	-ICP0, ICP4, ICP27	+	+	+++	ND	[29]
						
	-Promoter, E6, E7 (HPV16)	+	+	+++	ND	[13]
						
	-E6, E7 (HPV16, 18)	+	-	+++	ND	[11]
HPV						
	-E6, E7 (HPV16)	+	-	++	ND	[12, 54]
						
	E7 (HPV6, HPV11)	+	-	++	ND	[55]
						
	-cccDNA	-	+	+++	+++	[14–16, 56–61]
						
HBV	-HBsAg	+	-	++	++	[17, 62]
						
	-S, X	+	-	++	++	[63]
						
HCV	-host factor, (CLDN1, OCLN, mir122)	+	-	+++	ND	[64–66]
						
	-BART	-	+	+++	ND	[19]
EBV						
	-BVRF1	+	-	++	ND	[67]
						
JCV	-T-antigen	+	+	+++	ND	[28]
						
	-LTR	+	+	+++	ND	[20–22, 25,26, 68,69]
						
	-LTR	+	-	++	ND	[40, 41]
						
HIV-1	-Gag/Pol	+	+	+++	ND	[25, 26]
						
	-Env, Tat, Rev	+	-	+++	ND	[26]
						
	-host factors (CCR5, CXCR4, SAMHD1)	+	-	++	ND	[70–75]
						
	-LTR/Gag D	-	+	++	++	[23]

ND: Not determined

+ indicates experiment performed,—indicates no experiment

Inhibitory Effect: +++: Strong; ++: Moderate

Another approach is engineering of new Cas9 variants that cleave at a site outside the target so mutations from NHEJ will not prevent binding and cleavage of Cas9 [76]; e.g., Cpf1 cleaves a region distal from target [77, 78]. Another possibility is to combine CRISPR/Cas9 with RNAi to apply a double assault. RNAi approaches exist for a number of viruses, including HIV-1 [43, 79], HBV [80], HCV [81], HPV [82, 83], JCV [84], and HSV [85]. For example, RNAi against HIV-1 is already reaching the clinic [43, 79]. Delivery and the production of escape mutants are a problem, but combination of RNAi and CRISPR/Cas9 may address these problems. Delivery of both could be performed at the same time, and, just as multiplexing gRNAs has advantages, so the combination of RNAi and CRISPR/Cas9 has advantages over either approach employed alone. Another advantage would be that reduction in viral replication rate by RNAi would be expected to reduce the viral replication rate and viral titer and, hence, render virus more susceptible to CRISPR/Cas9 cleavage [68]. Thus, the combined use of an agent such as RNAi or a small molecule antiviral that slows viral replication may enhance the efficacy of CRISPR. HIV-positive subjects eligible for treatment with CRISPR/Cas9 would also use combined antiretroviral therapy, which would significantly improve the efficacy of CRISPR/Cas9 alleviating concerns related to resistance conferred by high rates of viral replication.

Finally, two other approaches have been applied that should not be affected by CRISPR/Cas9-generated escape mutants. In the case of HIV-1, CRISPR/Cas9 can disrupt cellular HIV coreceptors, CCR5 and CXCR4 [71, 86]; cells become refractory to HIV infection, and CRISPR/Cas9 would not be expected to generate escape mutants. Another approach is the use of Cas9 mutants deficient in endonuclease activity with a transcriptional activation domain (VP64) to target the HIV promoter region in a "shock and kill" strategy [87, 88]. Because the mutant Cas9 has no nuclease activity, there is no possibility of escape mutants being generated.

In conclusion, the possibility of generating escape mutants is a significant consideration when using CRISPR/Cas9 against any virus. This underlines the importance of adopting an appropriate design for gene editing strategies, but it does not detract from the promise of this approach in the treatment of human viral diseases.
